# Machine Learning Approach for Cardiovascular Death Prediction among Nonalcoholic Steatohepatitis (NASH) Liver Transplant Recipients

**DOI:** 10.3390/healthcare12121165

**Published:** 2024-06-08

**Authors:** Yasin Fatemi, Mohsen Nikfar, Amir Oladazimi, Jingyi Zheng, Haley Hoy, Haneen Ali

**Affiliations:** 1 Department of Industrial and Systems Engineering, Auburn University, Auburn, AL 36849, USA; yzf0024@auburn.edu (Y.F.); mzn0042@auburn.edu (M.N.); azo0048@auburn.edu (A.O.); 2 Department of Mathematics and Statistics, Auburn University, Auburn, AL 36849, USA; jingyi.zheng@auburn.edu; 3 College of Nursing, The University of Alabama in Huntsville, Huntsville, AL 35805, USA; haley.hoy@uah.edu; 4 Health Services Administration Program, Auburn University, Auburn, AL 36849, USA

**Keywords:** liver transplant, NASH, cardiovascular, machine learning, UNOS

## Abstract

Cardiovascular disease is the leading cause of mortality among nonalcoholic steatohepatitis (NASH) patients who undergo liver transplants. In the present study, machine learning algorithms were used to identify important risk factors for cardiovascular death and to develop a prediction model. The Standard Transplant Analysis and Research data were gathered from the Organ Procurement and Transplantation Network. After cleaning and preprocessing, the dataset comprised 10,871 patients and 92 features. Recursive feature elimination (RFE) and select from model (SFM) were applied to select relevant features from the dataset and avoid overfitting. Multiple machine learning algorithms, including logistic regression, random forest, decision tree, and XGBoost, were used with RFE and SFM. Additionally, prediction models were developed using a support vector machine, Gaussian naïve Bayes, K-nearest neighbors, random forest, and XGBoost algorithms. Finally, SHapley Additive exPlanations (SHAP) were used to increase interpretability. The findings showed that the best feature selection method was RFE with a random forest estimator, and the most critical features were recipient and donor blood type, body mass index, recipient and donor state of residence, serum creatinine, and year of transplantation. Furthermore, among all the outcomes, the XGBoost model had the highest performance, with an accuracy value of 0.6909 and an area under the curve value of 0.86. The findings also revealed a predictive relationship between features and cardiovascular death after liver transplant among NASH patients. These insights may assist clinical decision-makers in devising strategies to prevent cardiovascular complications in post-liver transplant NASH patients.

## 1. Introduction

Acute liver failure (ALF) is a serious condition that can develop quickly. This condition can result from viral infections such as hepatitis [1,2,3] or liver damage caused by drugs [4]. Poor oxygen supply can also be a common risk factor due to cardiac, circulatory, or respiratory failure [5]. ALF can lead to severe complications affecting various organs, including cardiovascular instability, increased susceptibility to infection, acute kidney injury, and edema [6].

According to the Centers for Disease Control and Prevention (CDC), liver disease has been diagnosed in approximately 4.5 million adults, which accounts for 1.8% of the US population. Sadly, it became the ninth leading cause of death in 2021. Additionally, the mortality rates of ALF increased from 40,265 in 2015 to 56,585 in 2021 [7].

When other treatments fail or when liver function is severely impaired, a liver transplant (LT) may become an option. In 2021, a record high of 9234 LTs were performed, of which 93.8% involved livers from deceased donors and 6.2% from living donors [8]. The same authors (i.e., [8]) report that patient survival rates for adults who underwent LT were 94.3% after six months and 86.7% after three years.

This study focuses on cardiovascular disease and mortality among nonalcoholic steatohepatitis (NASH) patients who have undergone LT. NASH, an inflammatory subtype of nonalcoholic fatty liver disease (NAFLD), is the fastest-growing reason for LT and affects 3% to 6% of the US population [9]. The present study utilizes machine learning (ML) models to predict cardiovascular death (CVD) among NASH patients after LT. Various studies have explored ML applications to predict post-transplant complications, mortality rates, and cardiovascular events in LT recipients with NAFLD [10,11]. However, our study aimed to predict CVD in LT patients. As previously highlighted, CVD is a predominant factor contributing to mortality in this patient population. Moreover, utilizing SHAP values, we identified the pivotal determinants influencing CVD. Notably, our findings demonstrate the superior performance of our approach compared to other prediction methods documented in the existing literature. The present study applies five ML algorithms and eight feature selection methods to predict CVD among NASH patients post-LT, using pre-transplant features from the United Network for Organ Sharing (UNOS) database.

## 2. Materials and Methods

### 2.1. Study Framework

**Figure 1:**
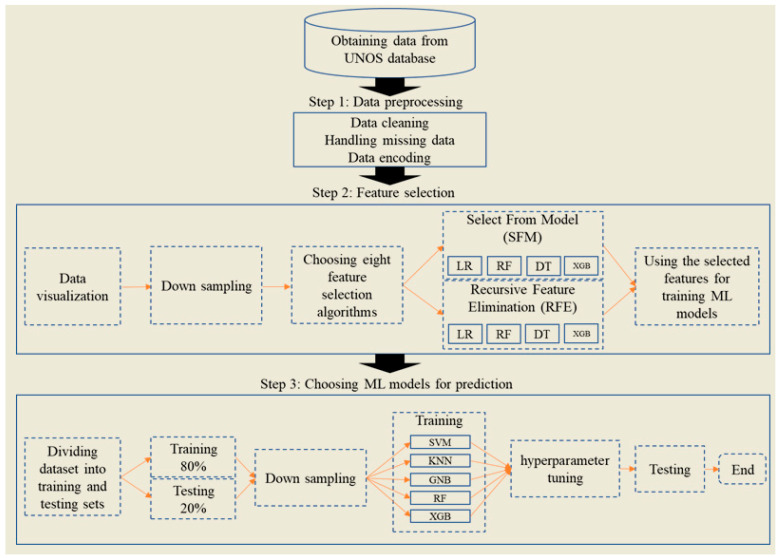
Overall study framework.

The framework of our current study comprises three main steps. First, for data preprocessing, we conducted key processes such as data cleaning, normalization, and transformation to prepare the data for in-depth analysis. The second step involved the application of various feature selection methods, which are crucial for identifying the most relevant features for our analysis. Finally, in the third step, we selected five widely recognized ML models for classification. This structured approach ensured a comprehensive analysis of the data, leading to accurate and reliable results (Figure 1).

### 2.2. Study Population and Data Collection

The STAR database provides LT recipient and donor data, which is submitted by Organ Procurement and Transplant Network (OPTN) members. Our study population included patients with NASH aged over 18 years who underwent LT between 1 October 1987, and 13 July 2022. Initially, 13,930 patients were identified. After removing invalid data, the number of recipient patients stood at 10,871.

The dataset comprises 1055 features associated with LT recipients and donors, spanning pre-, intra-, and post-transplant stages. Our study focused on pre-transplant predictors; therefore, intra- and post-transplant data were excluded. This exclusion resulted in the removal of 587 features from our analysis. The recipient data includes demographic details (gender, age, BMI, race, income level) and clinical and laboratory information. Similarly, the donor data contains demographic, clinical, and laboratory details. Fifteen features were eliminated due to their lack of predictive power, including predictors such as patient ID number. In the final step, 45 invariant features were removed, as they did not capture new information [12,13]. After this comprehensive data refinement, 92 features remained for further analysis, as detailed in Supplementary Table S1.

### 2.3. Outcomes

This study’s primary outcome of interest is the occurrence of CVD following LT. CVD may result from various causes, including cardiovascular problems, such as arrhythmia, arterial/pulmonary embolism, congestive heart failure, myocardial infarction, and cardiac arrest, as well as cerebrovascular issues, such as embolic and hemorrhagic strokes [14,15,16].

In our dataset, there were 449 observations of patients who died from cardiovascular causes (Class 1), compared to 8261 observations of patients who were alive (Class 0) and 2161 observations of patients who had died due to non-cardiovascular causes (Class 2). Several studies have demonstrated that balanced data sets improve the overall classification performance of several base classifiers as compared to imbalanced data [17,18]. The down-sampling method was employed to avoid issues caused by imbalanced data [19]. Specifically, 449 observations were randomly selected from Classes 0 and 2 and combined with the Class 1 patients, resulting in a balanced dataset with a sample size of 1347 patients.

### 2.4. Model Development

Recent improvements in ML have significantly impacted healthcare, particularly in clinical applications such as disease diagnosis. The ML algorithms utilized in this study include decision tree (DT), support vector machine (SVM), random forest (RF), XGBoost (XGB), K-nearest neighbors (KNN), and Gaussian naïve Bayes (GNB). A total of 92 features were selected for analysis after applying exclusion criteria. These ML techniques vary in their methodologies: SVM projects the data in a high-dimensional feature space, where data points are not linearly separable [20]; the K in KNN refers to the number of nearest data points in the training test based on their similarity [21]; DT is a popular tree-based method that divides the predictor space into a few regions and defines a set of splitting rules to make predictions based on the trees [22]. Similarly, RF is a tree-based ensemble method that combines many DTs to improve predictions by reducing the variance [23]. LR predicts binary outcomes [22]; XGB is an improved tree-based method that iteratively corrects mispredictions [24]; and GNB employs Gaussian probability distribution assumptions [25,26].

This study applied two feature selection methods, recursive feature elimination (RFE) and select from the model (SFM), to enhance the robustness of ML models. RFE was chosen for its proficiency in effectively eliminating redundant and weak features while preserving independent ones, thereby ensuring robust performance [27]. The advantage of employing SFM lies in its capacity to set a specific threshold value, which acts as a boundary differentiating between features to be retained and those to be eliminated [28]. Combining these methods with four ML algorithms (i.e., LR, RF, DT, and XGB) resulted in eight distinct feature selection approaches. Each feature’s importance was determined by its selection frequency across various methods. All selected features, irrespective of their selection frequency, were included in the ML training process. This led to two groups of features: one selected individually by each feature selection method and another comprising all features selected at least once, totaling 20 unique features for the prediction model.

### 2.5. Statistical Analysis

To test the statistical differences between the three classes, we used the Kruskal–Wallis test for continuous variables, and for categorical variables, we applied the chi-square test. We determined the significance of the differences between the groups by analyzing the *p*-value, which was adjusted based on the false discovery rate for multiple tests.

### 2.6. ML Model Interpretation

In this study, the SHAP value, a concept from cooperative game theory, was used to interpret the best ML model [29]. SHAP values are typically employed to determine the significance of input values in ML methods. They illustrate the contribution of individual features to the overall prediction.

SHAP values were calculated for each feature across different patient classes (alive, CVD, and non-CVD), providing insight into how each feature influenced the model’s decision-making process. The magnitude of a SHAP value indicates the importance of a feature, while its sign shows the direction of impact on the prediction (positive or negative). This approach allowed for a better interpretation of the features’ roles in the final model. Using this approach, we were able to determine how important each feature is and how it affects the survival rate.

### 2.7. Performance Evaluation

The following performance evaluation metrics were utilized in this study: accuracy, sensitivity, precision, F1 score, and area under the receiver operating characteristics curve (AUROC). The most prevalent metric, AUROC, helps minimize misclassification costs [30]. A perfect AUROC value is 1, while a value of 0.5 indicates a performance equal to random selection. The other performance criteria are defined as follows:

$$\mathrm{Accuracy}=\frac{TP+TN}{TP+TN+FP+FN}$$

$$\mathrm{Sensitivity}=\frac{TP}{TP+FN}$$

$$\mathrm{Precision}=\frac{TP}{TP+FP}$$

$$F1\;\mathrm{score}=\frac{2\times TP}{2\times TP+FP+FN}$$

To compare ML methods, five-fold cross-validation and 100 bootstrap samples were performed for each run (500 in total). Regarding the hyperparameter tuning of the ML models, a grid search with five-fold cross-validation was used (for KNN: n_neighbors, weights, metric; for GNB: var_smoothing; for RF: max_depth, max_features, min_samples_leaf, min_samples_split, n_estimators; for XGB: max_depth, n_estimators, nthread, objective; for SVM: kernel, C, gamma were optimized). Meanwhile, 80% of the data were used to train the initial ML models, and the remaining 20% were randomly chosen to serve as an independent test set.

## 3. Results

Table 1 shows the characteristics of the recipient and donor participants. Among the 10,871 recipients, 76% (*n* = 8261) were alive, 4.1% (*n* = 449) had died of CVD, and 19.9% (*n* = 2161) had died of other causes. The average age of the recipients was 58.91 years. Furthermore, 47.46% (*n* = 5160) of the patients were female, and 85.86% (*n* = 9) differed significantly in terms of age. On average, patients in the CVD group were older (60.85 years) than the non-cardiovascular (60.07 years) and living (58.50 years) patients. Of the patients, 85.86% (9334) were non-Hispanic, and 14.14% (1537) were Hispanic. Meanwhile, the average BMI in the non-CVD death group was lower at 32.25 than in the CVD group and the alive group, which had average BMIs of 32.91 and 32.80, respectively. Among the three groups, there were also significant differences regarding ethnicity, type of diabetes albumin, international normalized ratio (INR), serum creatinine, and serum sodium levels. Regarding the donors, the average age was 42.61 years, and 43.13% (*n* = 4363) were female.

### 3.1. Feature Importance Outputs

In Table 2, the features selected by each method are presented. Among them, the recipient’s blood type was the only feature chosen unanimously by all methods. Donor blood type, recipient height, and actual year were selected six times, while the donor’s home state was selected five times. The features of age, recipient dialysis, recipient bilirubin, and recipient weight were each selected three times. Deceased donor cardiac arrest, recipient serum creatinine, donor hematocrit level, MELD score, and recipient state of residency were selected twice. Finally, total cold ischemic time, recipient albumin level, recipient INR, recipient serum sodium level, and recipient work for income were each selected once.

### 3.2. ML Methods’ Performance

**Figure 2:**
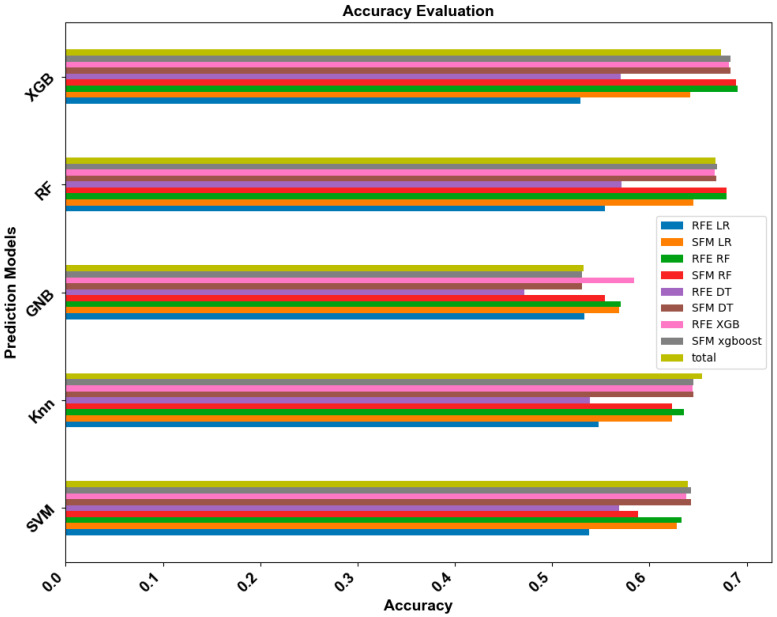
ML model accuracy for the feature selection methods.

Figure 2 presents the accuracy values for the various prediction methods applied to the ML models. In the SVM model, the application of SFM to both DT and XGB resulted in an identical accuracy of 0.6427. The highest accuracy achieved by the GNB model, at 0.5840, was achieved when RFE was applied alongside XGB. In the RF model, the best accuracy, 0.6794, was attained identically by both the RFE RF and SFM RF feature selection techniques. The highest accuracy value for the XGB model was 0.6909, achieved when the RFE was paired with RF for feature selection. As highlighted in Figure 2, the combination of XGB as the prediction model with RFE RF as the feature selection approach resulted in the most accurate outcomes. This combination also outperformed others in terms of the F1 score, precision, and sensitivity, as detailed in Supplementary Figures S1–S3.

**Figure 3:**
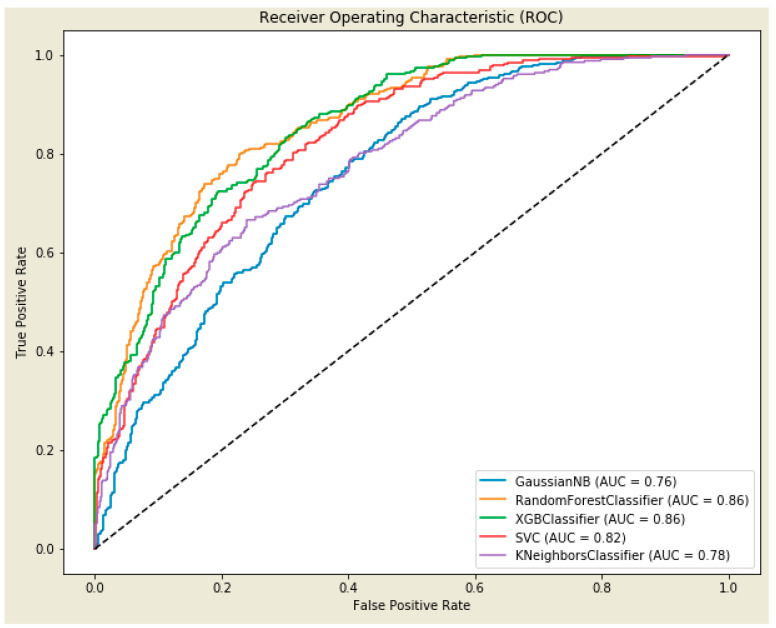
AUROC for predicting ML methods with the RFE RF feature selection method.

Figure 3 displays the AUROC curves for various ML predictions, all utilizing RFE RF for feature selection. The combination of XGB as the prediction model and RFE RF for feature selection emerged as the top performer, achieving an AUROC of 0.86. This score is close to the highest observed AUROC of 0.87 among all methods presented in Supplementary Figures S4–S11). Consequently, the XGB model, coupled with the features selected by RFE RF, was chosen for in-depth analysis and further discussion. This decision was based on XGB’s superior accuracy and competitively high AUROC score, which indicates its efficacy as a predictive model in the given context. Using the RFE RF method, the following features were selected: recipient blood type, actual year of the registrant, donor blood type, donor home state, recipient serum creatinine, recipient state of residency, and BMI.

### 3.3. SHAP Values

**Figure 4:**
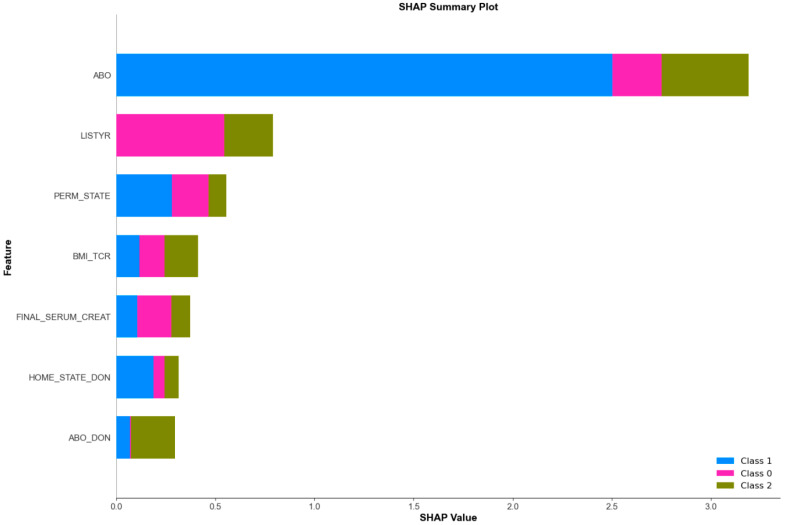
Importance of each feature for each class. ABO: recipient’s blood type, LYSTYR: recipient’s transplantation year, PERM_STATE: recipient’s permanent state of residence, BMI_TCR: recipient’s body mass index serum creatinine level, FI-NAL_SERUM_CREAT: recipient’s serum creatinine level, HOME_STATE_DON: donor’s geographic location, ABO_DON: donor’s blood type.

Figure 4 presents a SHAP summary plot, which ranks the features by their level of importance in the prediction of different outcome classes in the model. At the top of the plot, the most essential feature is the recipient’s blood type, which is particularly influential in predicting CVD, as evidenced by the prevalent blue section in its corresponding bar. The colors assigned within the plot—pink for patients who are alive, blue for CVD, and green for non-CVD—indicate the predictive significance of each feature for each class. The length of each bar signifies the strength of the feature’s influence on the model’s predictions for the respective classes.

**Figure 5:**
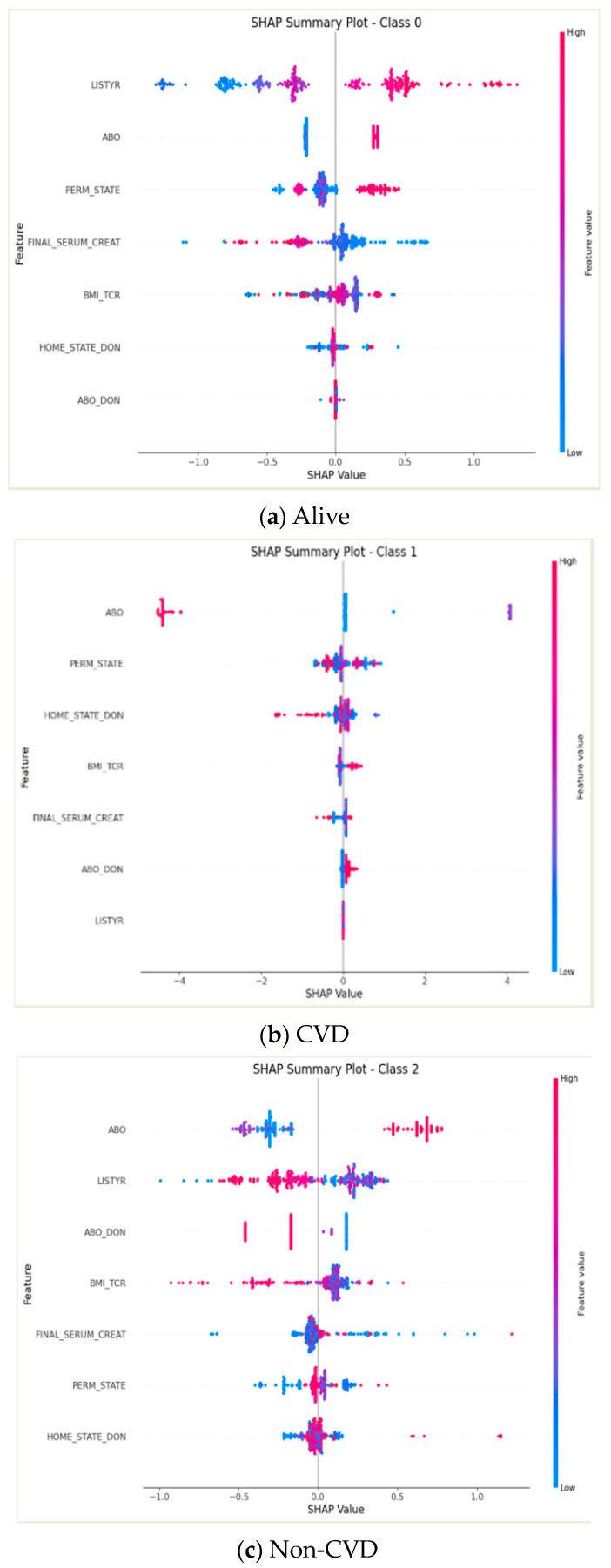
Feature importance among different classes. ABO: recipient’s blood type, LYSTYR: recipient’s transplantation year, PERM_STATE: recipient’s permanent state of residence, BMI_TCR: recipient’s body mass index serum creatinine level, FI-NAL_SERUM_CREAT: recipient’s serum creatinine level, HOME_STATE_DON: donor’s geographic location, ABO_DON: donor’s blood type.

Figure 5 presents the significance of various features in the model, as determined by SHAP values. Each dot’s position along the *x*-axis reflects the feature’s influence, where a positive SHAP value suggests an enhancement in the model’s target prediction. In contrast, a negative value implies a reduction. The dot’s hue indicates the magnitude of the feature’s value: warmer tones denote higher feature values, whereas cooler tones suggest lower ones. The vertical positioning of the features on the *y*-axis conveys their level of importance, with features placed higher on the axis being more influential in predicting the target variable. This particular figure illustrates the outcomes using the XGB model, with feature selection conducted via the RFE RF method.

As shown in Figure 5a, the transplantation year emerged as the primary predictor of the survival outcome of the recipients. This was followed in significance by factors such as the recipient’s ABO blood type, permanent state of residence, levels of serum creatinine, and BMI, as well as the donor’s geographic location and blood type. The SHAP summary plot analysis suggests that elevated serum creatinine levels—visualized through a predominance of red dots skewed to the left on the SHAP value axis—correlate with a reduced survival probability. This graphical representation underscores the negative correlation between higher serum creatinine levels and recipient survival, a critical insight within the domain of transplant medicine analytics.

From Figure 5b, it is understood that within the predictive model for CVD post-transplantation, the recipient’s ABO blood type holds the highest predictive weight. It is more influential than the recipient’s state of residence, BMI, and serum creatinine levels; the donor’s state of residence and donor’s blood type; and the transplantation year. Moreover, the analysis shows that the donor’s geographical origin plays a role in CVD outcomes, with the SHAP values indicating a trend in which donors from certain states are associated with a lower risk of CVD in recipients. This is visually represented by a cluster of red dots skewed to the left for the donor state of residence feature. For example, the model suggests that donors from states later in the alphabet, such as Washington, may correspond to a decreased CVD risk in the predictive modeling. In Figure 5c, the SHAP summary plot identifies the recipient’s ABO blood type as the predominant variable in predicting non-cardiovascular mortality. This is followed by the year of the transplant, the ABO blood type of the donor, and the recipient’s BMI, serum creatinine levels, and state of residence, with the donor’s state of residence also playing a role. The plot indicates that an increase in the recipient’s BMI correlates with a higher incidence of non-cardiovascular mortality.

## 4. Discussion

NASH is a liver disease that occurs due to chronic inflammation and fat accumulation and can eventually lead to cirrhosis. Liver transplantation is a definitive treatment for end-stage liver disease, including complications from conditions such as NASH-related cirrhosis. However, several factors can impact post-transplant outcomes, such as cardiovascular problems. To implement timely therapies and improve patient outcomes, the detection and prediction of CVD after LT in NASH patients are essential. The present study used ML techniques with extensive data preparation to clean the STAR dataset. The findings have substantial value in terms of detecting novel risk factors for CVD among NASH patients.

### 4.1. Comparison with Previous Studies

While other studies have focused on CVD after LT in general [10,31], this study focused on NASH patients, as CVD after LT is one of the most common causes of death for this group. Several predictive analytical procedures were integrated to maximize prediction model performance, and substantial data preparation was conducted as a preprocessing strategy for sophisticated classification methods. Several studies have used various ML techniques, such as RF, XGB, SVM, and LR, in their analyses [10,11,32]. In the present study, the best prediction model, XGB, had an AUROC value of 0.8663, which showed a superior ability to predict CVD after LT than other studies in the area [10,11]. According to the present study’s selected model (XGB), the predictors of CVD among NASH patients were, in order of importance, the recipient’s blood type and state of residence, the donor’s state of residence, the recipient’s BMI and serum creatinine level, the donor’s blood type, and the year of transplantation.

Several researchers have identified various factors contributing to cardiovascular problems in LT patients that differ from the features we found to be significant. Kim et al. highlighted obesity, dyslipidemia, hypertension, and diabetes mellitus as significant risk factors. Albeldawi et al. found older age at transplantation, being male, new-onset diabetes mellitus, hypertension, and mycophenolate mofetil application to be major cardiovascular risk factors [33]. Dec et al. concluded that only older age and pre-existing cardiac disease had a significant effect on serious cardiovascular problems [34]. Fussner et al. identified age, left ventricular hypertrophy, diabetes, ejection fraction < 60%, hypertension, prior cardiovascular disease, glomerular filtration rate < 60 mL/minute, and serum troponin (TN) > 0.07 ng/mL as the main contributors to cardiovascular issues in LT patients [35].

However, our findings align with those of the following authors who highlighted the importance of blood type and BMI as predictors of post-transplant outcomes. For instance, studies by Chen et al. (2020) and Jain et al. (2021) identified blood type as a significant predictor of cardiovascular events in transplant patients [10,36]. Additionally, the importance of serum creatinine as a predictor of cardiovascular outcomes is consistent with findings from studies by Bagheri et al. (2019) and Wannamethee et al. (1997) [37,38].

Our study extends the current literature by integrating SHAP values to interpret the model’s predictions, providing a clearer understanding of how individual features influence the risk of CVD. This interpretability is crucial for clinical decision-making and improving patient outcomes. Given the variability in factors identified by different researchers, our findings should be used by physicians as guidelines for further clinical analyses to verify these results.

### 4.2. Important Features

#### 4.2.1. Blood Type

One clinical data parameter that strongly predicted CVD was blood type. Previous studies have shown that the ABO blood group plays a significant role in hemostasis by influencing the plasma concentrations of the von Willebrand factor (VWF) and coagulation factor VIII (FVIII). VWF and FVIII levels in non-O blood groups are approximately 25% higher than in the O blood group [39,40] due to the positive effect of the addition of A and B antigens to existing VWF H oligosaccharides by specific glycosyltransferase enzymes [41]. Several inflammatory cytokines, such as tumor necrosis factor alpha, soluble intercellular adhesion molecule 1, E-selectin, P-selectin, and interleukin-6, as well as cholesterol levels, have been suggested as the most likely mechanisms for the association between the ABO blood group and CVD [42,43,44,45]. Meanwhile, a study of a large population of Italian blood donors evaluated the clinical impact of cardiovascular disease. A close link between non-O blood type and cardiovascular risk has also been reported [46,47,48].

Although the ABO blood group has been shown to have a relationship with blood coagulation, ischemic heart disease, and deep vein thrombosis [36,49], as a non-modifiable risk factor, it may lack clinical significance. LT patients with non-O blood types and their clinicians might consider it a motivator to be more vigilant about CVD prevention in this cohort. In contrast, BMI and serum creatinine are modifiable risk factors that can be addressed pre- and post-transplant. Current research suggests that clinicians managing pre-transplant waiting lists must be mindful of BMI and creatinine management. Furthermore, post-transplant clinicians should consider more stringent goals of care for patients with non-O blood types.

#### 4.2.2. Body Mass Index

The relationship between BMI and cardiovascular problems found in this study is consistent with the findings of many papers that studied this problem [50,51,52,53,54,55,56]. It is worth noting that people with the same BMI can have different fitness levels, and BMI alone cannot be a good measure of fitness. Nevertheless, BMI can be a relevant factor in predicting CVD among NASH patients who have undergone an LT, and many factors, such as metabolic syndrome, can explain the causal effect of a high BMI on CVD. Cardiovascular risk has been found to increase with abdominal obesity, dyslipidemia, insulin resistance, type 2 diabetes, and hypertension [57,58,59,60]. Obesity, defined by the WHO as a BMI equal to or above 30 kg/m^2^ [61], could also contribute to the development of other cardiovascular risks caused by chronic low-grade inflammation resulting from obesity [62,63].

It is important to acknowledge the limitations of BMI in measuring body fat distribution and in differentiating between fat and muscle mass [64,65]. Nonetheless, as a screening tool, BMI provides valuable insights into specific health conditions [66,67].

#### 4.2.3. Serum Creatinine

Kidney disease has been associated with cardiovascular events and death [68,69,70], and some studies have reported that kidney disease has an effect on cardiac events after LT [71].

Some previous studies have demonstrated a positive association between serum creatinine levels and CVD [37,38,72]. However, [73] found that this relationship was partially impacted by blood lipids, BMI, blood pressure, high-sensitivity C-reactive protein (hs-CRP), blood glucose, and other factors. The findings of our study support those of [73] by indicating that a low serum creatinine level might cause CVD, highlighting a variation in the effect of serum creatinine.

#### 4.2.4. State of Residency

Access to healthcare may differ depending on variables including socioeconomic status, geographic location, insurance coverage, and healthcare system structures. Lack of healthcare access can result in delayed diagnosis, inadequate management of cardiovascular risk factors, and decreased medication adherence, all of which can increase the risk of CVD [74,75,76].

Our findings regarding the state of residency and access to care support the need for outreach clinics for transplantation. Moreover, the need for regulatory consideration of geographic locations for transplant centers is critical because UNOS is currently considering a restructuring. UNOS (2022) recently expressed a desire to broaden equity in access to transplant healthcare, making the present study’s findings timely for the field of transplantation.

### 4.3. Strengths and Limitations

One of the strengths of this study is the comprehensive dataset used, encompassing a large number of patients and a wide range of features. The application of advanced ML techniques and the use of SHAP values for model interpretation are also notable strengths.

However, the study had a few limitations to consider. First, the research was retrospective in nature and was based on information from the UNOS database, which is prone to bias and inaccurate information. Despite demonstrating great accuracy, our model still has space for improvement, especially in handling datasets that are not evenly distributed. Further research could examine different methods for improving models and balancing data.

### 4.4. Future Research Directions

To improve the model’s performance, future research should concentrate on verifying our findings in prospective studies and investigating the incorporation of extra clinical variables. Furthermore, examining the biological mechanisms that underlie the associations between the discovered predictors and cardiovascular outcomes may yield a more profound understanding and improve the precision of predictions.

To ensure the generalizability and robustness of these prediction models across a range of patient cohorts, more research might examine the use of these models in various settings and populations.

## 5. Conclusions

The use of ML techniques in healthcare has become popular because these methods can enable faster and more accurate discovery of patterns in healthcare data. However, most ML techniques do not address the important aspect of interpretability in their approach [77]. One of the main contributions of this work is its application of an interpretable ML approach for predicting. The present study used five ML algorithms to predict CVD among post-LT NASH patients. The sample size was 10,873, and the number of predictors after data cleaning and preprocessing was 92. The XGB prediction model with RFE RF feature selection outperformed the other models. The best features in predicting CVD after LT among NASH patients were donor and recipient state of residency, recipient serum creatinine, recipient BMI, and donor and recipient blood type. We presented graphs created based on SHAP values to make our ML technique interpretable, to demonstrate how it makes predictions, and to indicate the most important features identified by the ML used. Clinicians could use this study’s findings to make the right decisions regarding therapeutic interventions for NASH patients.

## Figures and Tables

**Table 1:** Baseline demographic characteristics according to class (0 = alive, 1 = cardiovascular death, and 2 = non-CVD).

Characteristics	All Recipients (*n* = 10,871)	Alive (*n* = 8261)	CVD (*n* = 449)	Non-CVD Death (*n* = 2161)	*p*-Value	Adjusted *p*-Value
**Recipient characteristics**						
**Age (years)**	58.91	58.50	60.85	60.07	< 0.001	<0.001 *
**BMI (kg/m)**	32.70	32.80	32.91	32.25	0.002	0.004 *
**Ethnicity**					<0.001	<0.001 *
Non-Hispanic	9334 (85.86%)	7010 (84.85%)	390 (86.86%)	1934 (89.50%)		
Hispanic	1537 (14.14%)	1251 (15.14%)	59 (13.14%)	227 (10.50%)		
**Gender**					0.470278	0.470278
Female	5160 (47.46%)	3939 (47.68%)	219 (48.77%)	1002		
Male	5711 (52.54%)	4322 (52.31%)	230 (51.22%)	1159		
**Blood Type**					0.257287	0.257287
O	4780 (43.97%)	3619 (43.80%)	202 (45.00%)	959 (44.38%)		
A	4037 (37.14%)	3033 (36.71%)	179 (40.00%)	825 (38.18%)		
B	1436 (13.21%)	1108 (13.41%)	57 (12.50%)	271 (12.55%)		
AB	600 (5.50%)	486 (5.88%)	11 (2.50%)	103 (4.76%)		
A1	14 (1.28%)	11 (0.14%)	0	3 (0.13%)		
A1B	2 (0.018%)	2 (0.03%)	0	0		
A2B	1 (0.009%)	1 (0.015%)	0	0		
A2	1 (0.009%)	1 (0.015%)	0	0		
**Diabetes**					<0.001 *	<0.00 *
No	4836 (44.48%)	3829 (46.35%)	161 (35.85%)	846 (39.14%)		
Type I	234 (2.15%)	133 (1.60%)	16 (3.56%)	85 (3.93%)		
Type II	5462 (50.24%)	4082 (49.42%)	252 (56.12%)	1128 (52.19%)		
Other type	105 (0.96%)	82 (0.99%)	4 (0.89%)	19 (0.87%)		
Unknown type	190 (1.74%)	109 (1.32%)	14 (3.14%)	67 (3.10%)		
Diabetes status unknown	44 (40%)	26 (0.32%)	2 (0.44%)	16 (0.74%)		
**Albumin (g/dL)**	3.11	3.13	3.13	3.05	<0.001 *	<0.001 *
**BILIRUBIN (μmol/L)**	7.67	7.63	8.57	7.63	0.03 *	0.05828
**INR**	1.97	1.99	1.98	1.89	<0.001 *	<0.001 *
**MELD score**	25.21	25.21	26.19	25.00	0.04	0.06
**SERUM CREAT (mg/dL)**	1.83	1.77	2.10	2.00	<0.001 *	<0.001 *
**SERUM SODIUM (mmol/L)**	135.12	134.99	135.22	135.60	<0.001 *	<0.001 *
**Donor characteristics**						
**Age (years)**	42.61	42.48	42.27	43.15	0.27	0.36
**Gender**					0.47	0.47
Female	4363 (40.13%)	3294 (39.87%)	177 (39.42%)	892 (41.28%)		
Male	6508 (59.86%)	4967 (60.13%)	272 (60.58%)	1269 (58.72%)		
Diabetes					0.43	0.43
No	9399 (86.46%)	7135 (86.64%)	389 (86.67%)	1875 (86.77%)		
Yes	105 (0.96%)	87 (0.010%)	5 (1.11%)	13 (0.60%)		
Unknown	1367 (12.58%)	1039 (12.57%)	55 (12.24%)	273 (12.63%)		
**Blood Type**					0.06	0.06
O	1588 (14.60%)	1193 (14.44%)	72 (16.03%)	323 (14.96%)		
A	2210 (20.32%)	1636 (19.80%)	98 (21.82%)	476 (22.02%)		
B	156 (1.44%)	123 (1.49%)	4 (0.89%)	29 (1.34%)		
AB	364 (3.35%)	293 (3.54%)	143 (3.11%)	57 (2.63%)		
A1	54 (0.49%)	47 (0.56%)	1 (0.22%)	6 (0.27%)		
A1B	165 (1.51%)	134 (1.62%)	2 (0.44%)	29 (1.35%)		
A2B	1307 (12.02%)	1020 (12.35%)	46 (10.24%)	241 (11.15%)		
A2	5027 (46.24%)	3815 (46.18%)	212 (47.21%)	1000 (46.27%)		

* Adjusted *p*-value < 0.05.

**Table 2:** Feature selection results.

Feature Number	Feature	RFE_LR	SFM_LR	SFM_RF	RFE_RF	RFE_DT	SFM_DT	SFM_XGB	RFE_XGB	Number of Time Selected
1	Recipient blood type	✓	✓	✓	✓	✓	✓	✓	✓	8
2	Recipient height	✓	✓			✓	✓	✓	✓	6
3	Actual year the registrant		✓	✓	✓		✓	✓	✓	6
4	Donor blood type	✓	✓		✓		✓	✓	✓	6
5	Donor home state			✓	✓		✓	✓	✓	5
6	Recipient bilirubin	✓	✓				✓			3
7	Age						✓	✓	✓	3
8	Recipient weight (kg)	✓	✓	✓						3
9	Recipient dialysis						✓	✓	✓	3
10	Donor hematocrit level			✓		✓				2
11	Recipient serum creatinine				✓	✓				2
12	Recipient state of residency			✓	✓					2
13	MELD score	✓	✓							2
14	Deceased donor cardiac arrest						✓	✓		2
15	BMI			✓	✓					2
16	Recipient serum sodium level					✓				1
17	Recipient work for income					✓				1
18	Recipient INR					✓				1
19	Recipient albumin level					✓				1
20	Total cold ischemic time			✓						1

## Data Availability

The data are publicly available on request.

## Supplementary Materials

The following supporting information can be downloaded at: https://www.mdpi.com/article/10.3390/healthcare12121165/s1, Figure S1. F1 score of ML models for different feature selection methods. Figure S2. Precision of ML models for different feature selection methods. Figure S3. Sensitivity of ML models for different feature selection methods. Figure S4. AUC ROC for predicting ML methods with SFM XGB feature selection. Figure S5. AUC ROC for predicting ML methods with RFE XGB feature selection. Figure S6. AUC ROC for predicting ML methods with SFM DT feature selection. Figure S7. AUC ROC for predicting ML methods with RFE DT feature selection. Figure S8. AUC ROC for predicting ML methods with SFM RF feature selection. Figure S9. AUC ROC for predicting ML methods with SFM LR feature selection. Figure S10. AUC ROC for predicting ML Methods with RFE LR feature selection. Figure S11. AUC ROC for predicting ML methods with all the selected features. Table S1. Feature Definition and Kruskal Wallis and Chi Square Test Results.

## Glossary

ALF	Acute Liver Failure
CVD	Cardiovascular Death
DT	Decision Tree
GNB	Gaussian Naïve Bayes
GFR	Glomerular Filtration Rate
KNN	K-Nearest Neighbors
LT	Liver Transplant
ML	Machine Learning
NAFLD	Nonalcoholic Fatty Liver Disease
OPTN	Organ Procurement and Transplant Network
RF	Random Forest
RFE	Recursive Feature Elimination
RCRI	Revised Cardiac Risk Index
SFM	Select From Model
SHAP	Shapley Additive Explanations
SVM	Support Vector Machine
UNOS	United Network for Organ Sharing
VWF	Von Willebrand Factor
XGB	XGBoost
