# Supplementary material for: Machine Learning Approach for Cardiovascular Death Prediction among Nonalcoholic Steatohepatitis (NASH) Liver Transplant Recipients
Source: Healthcare (Basel). 2024 Jun 8;12(12):1165. doi: 10.3390/healthcare12121165 (PMC11202858; doi:10.3390/healthcare12121165)
Supplement: Supplementary file 1 [file healthcare-12-01165-s001.zip › healthcare-2987669-supplementary.pdf]

## Supplementary:

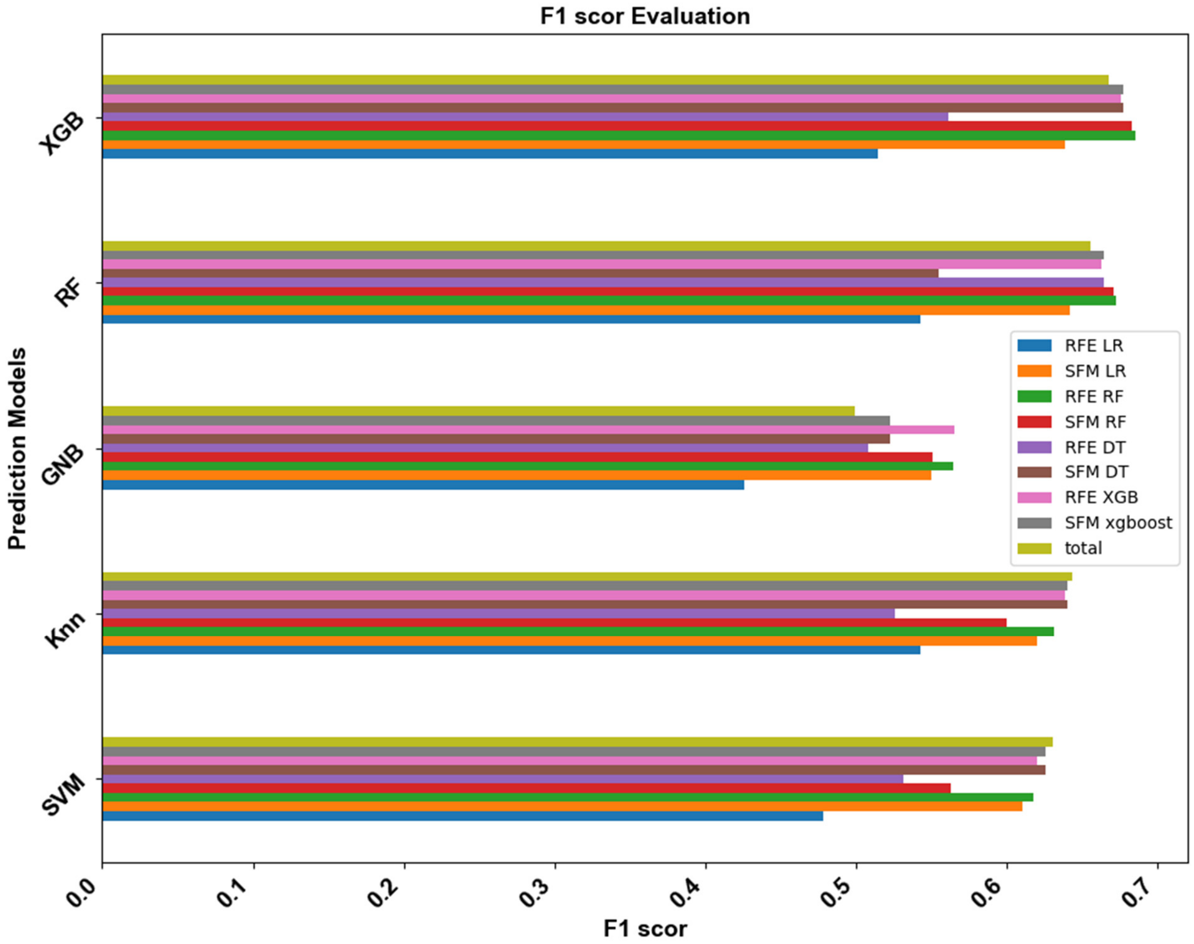


**Figure S1.** F1 score of ML models for different feature selection methods

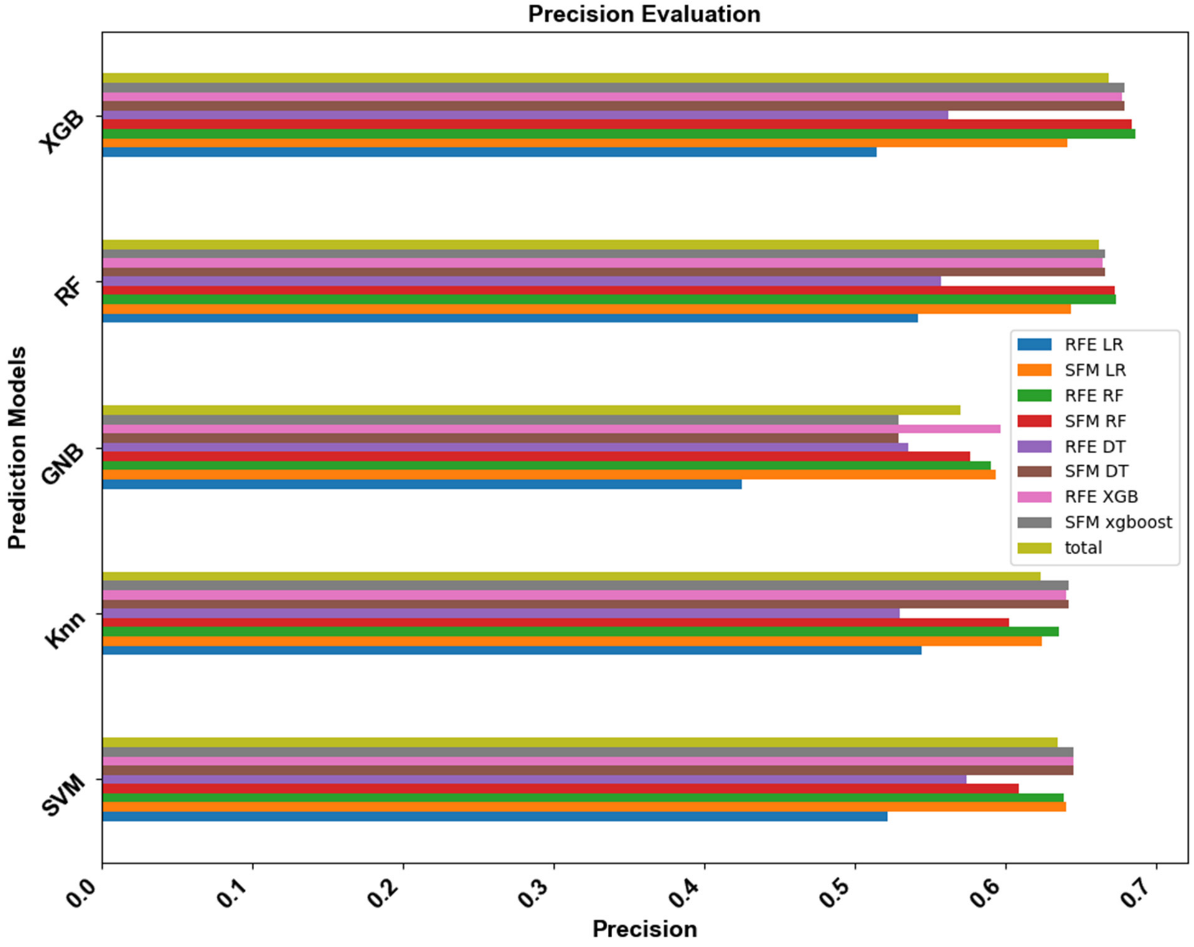


**Figure S2.** Precision of ML models for different feature selection methods

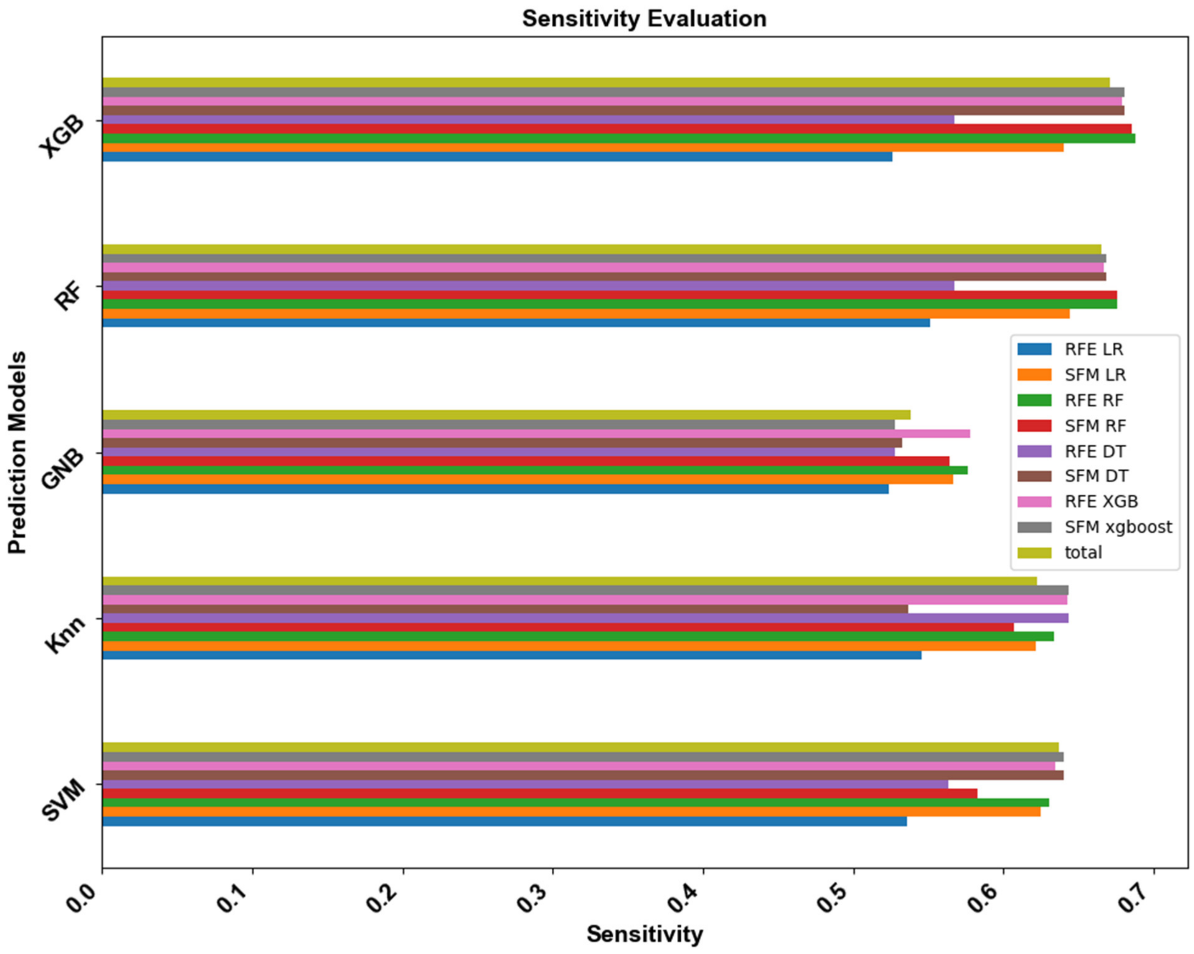


**Figure S3.** Sensitivity of ML models for different feature selection methods

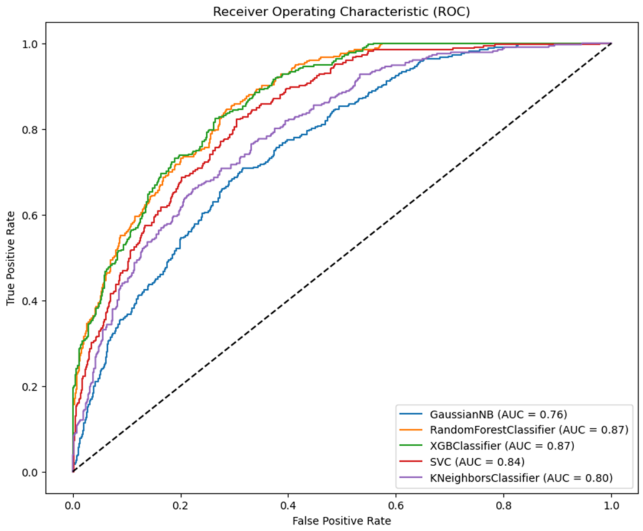


**Figure S4.** AUC ROC for predicting ML methods with SFM XGB feature selection.

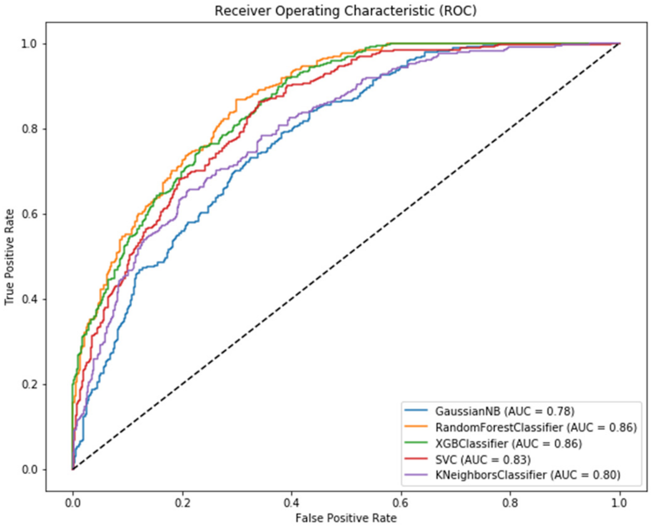


**Figure S5.** AUC ROC for predicting ML methods with RFE XGB feature selection

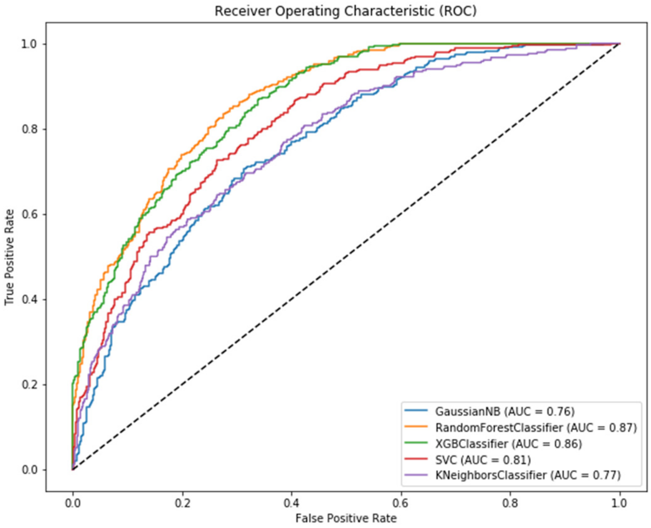


**Figure S6.** AUC ROC for predicting ML methods with SFM DT feature selection

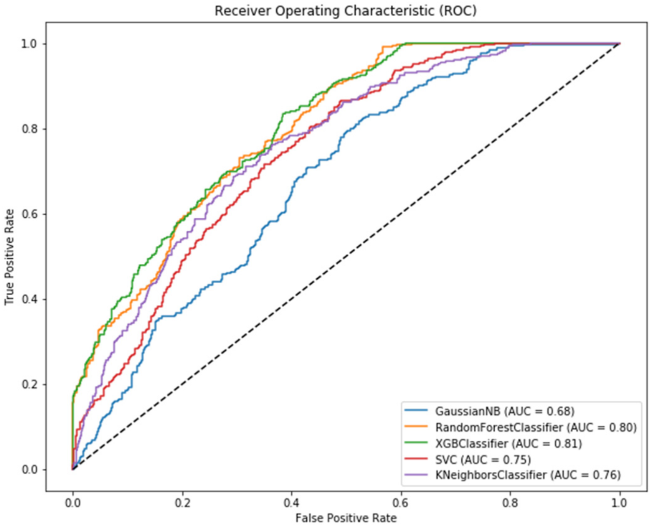


**Figure S7.** AUC ROC for predicting ML methods with RFE DT feature selection

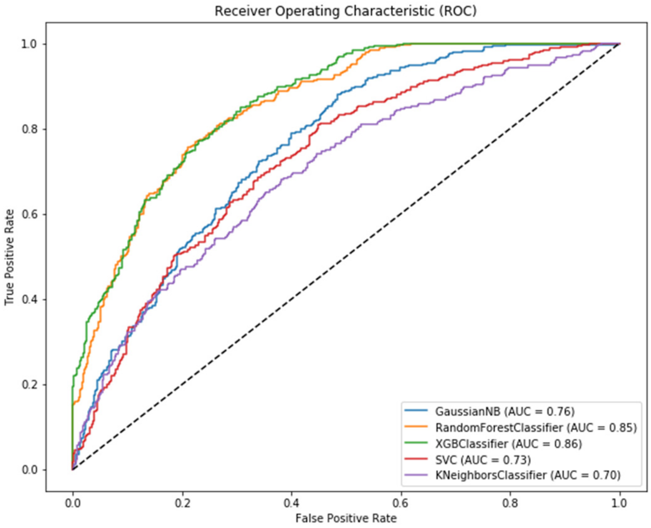


**Figure S8.** AUC ROC for predicting ML methods with SFM RF feature selection

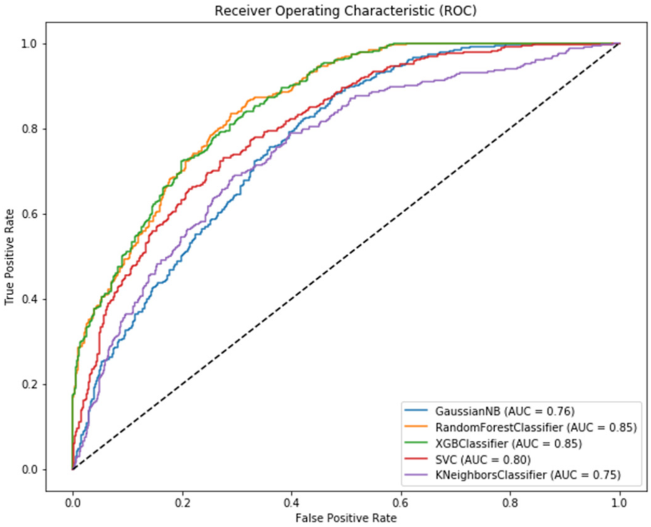


**Figure S9**. AUC ROC for predicting ML methods with SFM LR feature selection

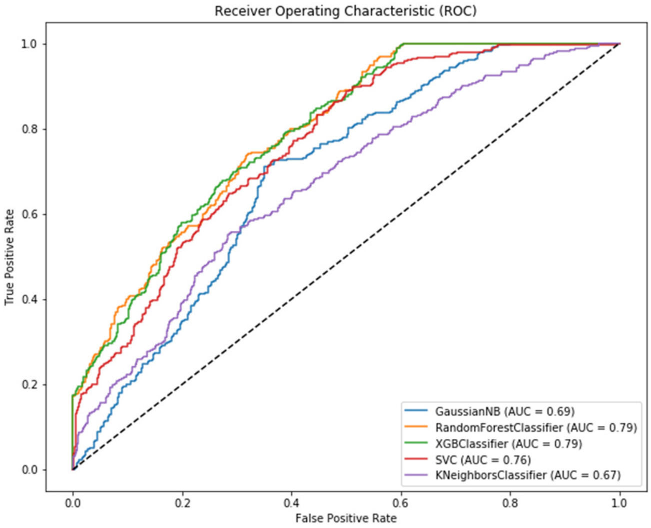


**Figure S10.** AUC ROC for predicting ML Methods with RFE LR feature selection

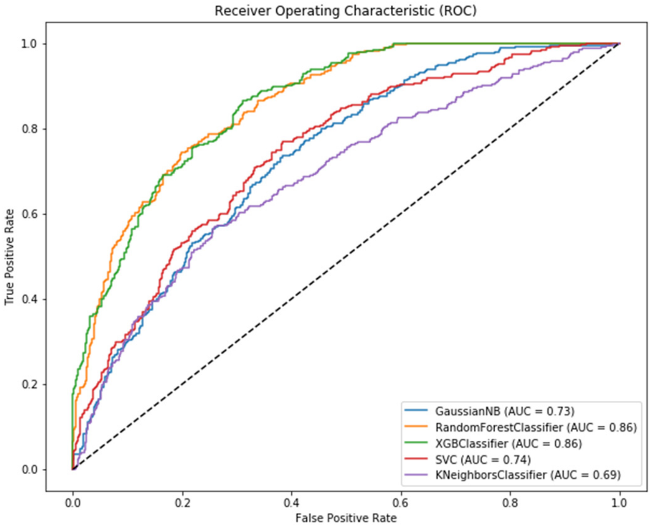


**Figure S11.** AUC ROC for predicting ML methods with all the selected features

**Table S1.** Feature Definition and Kruskal Wallis and Chi Square Test Results

| Features | description | 0 | 1 | 2 | p-value | adjusted p-value |
|---|---|---|---|---|---|---|
| ABO | recipient blood group @ registration | 4.168139 | 4.033408 | 4.098565 | 0.944161 | 0.944161 |
| ABO_DON | donor blood type | 4.411572 | 4.280624 | 4.312818 | 0.668028 | 0.717775 |
| AGE | recipient age (yrs) | 58.50176 | 60.85078 | 60.07034 | 1.12E-20 | 1.61E-19 |
| AGE_DON | donor age (yrs) | 42.48941 | 42.2784 | 43.15271 | 0.279065 | 0.366046 |
| ALCOHOL_HEAVY_DON | ddr heavy alcohol use (heavy= 2+ drinks/day) (y/n/u) | 0.348142 | 0.327394 | 0.31143 | 0.057159 | 0.08747 |
| ANTIHYPE_DON | deceased donor-antihypertensives w/in 24 hrs pre-cross clamp | 0.603075 | 0.552339 | 0.548357 | 0.031793 | 0.058383 |
| ARGININE_DON | deceased donor-was donor given arginine vasopressin within 24 hrs pre cross clamp? | 1.279143 | 1.260579 | 1.176307 | 5.92E-05 | 0.000171 |
| BLOOD_INF_DON | deceased donor-blood as infection source | 0.115119 | 0.095768 | 0.090699 | 0.003381 | 0.007715 |
| BMI_TCR | bmi at listing | 32.80865 | 32.91552 | 32.25073 | 0.001929 | 0.004532 |
| BUN_DON | deceased donor-terminal blood urea nitrogen | 26.40436 | 23.35584 | 22.96525 | 1.95E-18 | 2.19E-17 |
| CARDARREST_NEURO | deceased donor-cardiac arrest post brain death | 0.072751 | 0.064588 | 0.066173 | 0.490795 | 0.550781 |
| CDC_RISK_HIV_DON | deceased donor - per phs, does the donor have risk factors for blood-borne disease transmission? | 0.428398 | 0.329621 | 0.336881 | 2.19E-06 | 7.36E-06 |
| CLIN_INFECT_DON | deceased donor-clinical infection (y,n) | 1.439172 | 1.280624 | 1.262379 | 1.04E-16 | 9.56E-16 |
| COD_CAD_DON | deceased donor-cause of death | 21.9678 | 26.42094 | 17.72929 | 7.27E-07 | 2.72E-06 |
| COLD_ISCH | total cold ischemic time (hours) | 6.108828 | 6.615167 | 6.530255 | 1.71E-10 | 1.15E-09 |
| CORONARY_ANGIO_DON | deceased donor coronary agiogram done (y/n) | 0.239438 | 0.198218 | 0.188339 | 9.48E-07 | 3.42E-06 |
| CREAT_DON | deceased donor-terminal lab creatinine | 1.736506 | 1.635457 | 1.629745 | 0.216073 | 0.290978 |
| DEATH_CIRCUM_DON | deceased donor-circumstance of death | 131.1703 | 156.9198 | 144.9264 | 0.340485 | 0.429862 |
| DEATH_MECH_DON | deceased donor-mechanism of death | 24.80123 | 34.46325 | 26.67515 | 7.81E-13 | 6.07E-12 |
| DGN_TCR | primary diagnosis at time of listing | 4191.065 | 4144.399 | 4152.883 | 5.86E-05 | 0.000171 |
| DIAB | recipient diabetes @ registration | 5.224791 | 6.750557 | 9.615456 | 1.25E-10 | 9.05E-10 |

| | | | | | | |
|---|---|---|---|---|---|---|
| DIABETES_DON | deceased donor-history of diabetes (y,n) | 0.262075 | 0.256125 | 0.258677 | 0.912497 | 0.926826 |
| DIAL_TX | dialysis prior week to transplant? | 0.184966 | 0.265033 | 0.231837 | 8.49E-09 | 4.29E-08 |
| ECD_DONOR | expanded donor per kidney allocation definition 1=yes | 0.267038 | 0.249443 | 0.291994 | 0.038291 | 0.064779 |
| EDUCATION | recipient highest educational level @ registration | 39.64593 | 54.74388 | 73.27395 | 0.189099 | 0.258095 |
| ETHNICITY | recipient ethnicity: 1=hispanic 0=non-hispanic | 0.151434 | 0.131403 | 0.105044 | 2.10E-07 | 8.18E-07 |
| EXC_CASE | active m/p except. case at removal or date of analysis (between 2/27/2002 - 6/28/2022) | 0.105798 | 0.093541 | 0.128644 | 0.005476 | 0.011523 |
| FINAL_ALBUMIN | most recent waiting list albumin or at removal if removed | 3.131767 | 3.134744 | 3.053429 | 1.30E-06 | 4.52E-06 |
| FINAL_ASCITES | most recent waiting list ascites or at removal if removed | 2.265222 | 2.387528 | 2.273484 | 0.001071 | 0.002639 |
| FINAL_BILIRUBIN | most recent waiting list bilirubin or at removal if removed | 7.639139 | 8.574677 | 7.63714 | 0.031159 | 0.05828 |
| FINAL_DIALYSIS_PRIOR_WEEK | most recent waiting list dialysis twice in prior week or at removal if removed | 0.184239 | 0.265033 | 0.230449 | 9.46E-09 | 4.55E-08 |
| FINAL_ENCEPH | most recent waiting list encephalopathy or at removal if removed | 1.866602 | 1.915367 | 1.931976 | 5.58E-05 | 0.000171 |
| FINAL_INR | most recent waiting list inr or at removal if removed | 1.991978 | 1.980913 | 1.891388 | 1.64E-08 | 7.20E-08 |
| FINAL_MELD_PELD_LAB_SCORE | most recent waiting list meld/peld lab score or at removal if removed(may be different than allocation score/endstat) | 25.21959 | 26.19376 | 25.00463 | 0.039592 | 0.064779 |
| FINAL_SERUM_CREAT | most recent waiting list serum creatinine or at removal if removed | 1.773691 | 2.100735 | 2.006182 | 1.20E-22 | 2.42E-21 |
| FINAL_SERUM_SODIUM | most recent waiting list serum sodium or at removal if removed | 134.996 | 135.2294 | 135.605 | 9.47E-06 | 3.08E-05 |
| FUNC_STAT_TCR | recipient functional status @ registration | 2018.874 | 2027.361 | 1998.531 | 0.690432 | 0.734038 |
| GENDER | recipient gender | 0.523181 | 0.512249 | 0.536326 | 0.470311 | 0.535376 |
| GENDER_DON | donor gender | 0.601259 | 0.605791 | 0.587228 | 0.471767 | 0.535376 |
| HEMATOCRIT_DON | ddr:hematocrit: | 29.39067 | 29.87305 | 30.1615 | 1.80E-08 | 7.57E-08 |
| HEP_C_ANTI_DON | deceased donor-antibody to hep c virus result | 0.114393 | 0.082405 | 0.050902 | 7.86E-09 | 4.18E-08 |
| HGT_CM_TCR | recipient height @ registration | 170.406 | 169.5702 | 170.6147 | 0.129436 | 0.184127 |
| HIST_CIG_DON | deceased donor-history of cigarettes in past @ >20pack yrs | 0.472098 | 0.579065 | 0.518741 | 0.011244 | 0.022267 |
| HIST_COCAINE_DON | deceased donor-history of cocaine use in past | 0.406367 | 0.374165 | 0.344285 | 0.003437 | 0.007715 |
| HIST_DIABETES_DON | deceased donor-history of diabetes, incl. duration of disease | 11.78816 | 12.35412 | 7.26099 | 0.734469 | 0.772723 |
| HIST_HYPERTENS_DON | deceased donor-history of hypertension | 0.761288 | 0.69265 | 0.757057 | 0.34645 | 0.431994 |
| HIST_OTH_DRUG_DON | deceased donor-history of other drug use in past | 0.921922 | 0.770601 | 0.792226 | 1.34E-08 | 6.13E-08 |

| | | | | | | |
|---|---|---|---|---|---|---|
| HISTORY_MI_DON | ddr history of prior mi | 0.097446 | 0.073497 | 0.092087 | 0.370338 | 0.456148 |
| HOME_STATE_DON | dr home state | 25.16233 | 26.11136 | 25.98889 | 0.039765 | 0.064779 |
| INIT_AGE | age in years at time of listing | 57.99988 | 60.23831 | 59.51411 | 7.37E-19 | 9.30E-18 |
| INIT_ALBUMIN | initial waiting list albumin | 3.087623 | 3.106682 | 3.065248 | 0.447861 | 0.519931 |
| INIT_ASCITES | initial waiting list ascites | 2.211112 | 2.289532 | 2.213327 | 0.05347 | 0.083084 |
| INIT_BILIRUBIN | initial waiting list bilirubin | 5.912373 | 6.145189 | 5.59894 | 7.16E-09 | 4.02E-08 |
| INIT_ENCEPH | initial waiting list encephalopathy | 1.818908 | 1.821826 | 1.845442 | 0.135006 | 0.189383 |
| INIT_HGT_CM | candidate height in cm at listing | 170.342 | 169.5307 | 170.5792 | 0.118529 | 0.17102 |
| INIT_INR | initial waiting list inr | 1.741277 | 1.662673 | 1.659866 | 2.17E-13 | 1.82E-12 |
| INIT_MELD_PELD_LAB_SCORE | initial waiting list meld/peld lab score(may be different than what used for allocation/initstat) | 21.66953 | 21.49889 | 21.0236 | 0.000981 | 0.002476 |
| INIT_SERUM_CREAT | initial waiting list serum creatinine | 1.563872 | 1.826169 | 1.773975 | 1.58E-21 | 2.65E-20 |
| INIT_SERUM_SODIUM | initial waiting list serum sodium | 135.3203 | 135.6682 | 135.7214 | 0.004876 | 0.010478 |
| INIT_STAT | candidate status at listing (excluding liver exception points after 6/28/2022) | 6227.253 | 6229.131 | 6223.753 | 0.000105 | 0.000296 |
| INIT_WGT_KG | candidate weight in kg at listing | 95.74149 | 95.32184 | 94.20894 | 0.093316 | 0.136593 |
| INOTROP_SUPPORT_DON | recipient life support type iv inotorpes @ registration | 0.95969 | 1.057906 | 1.048126 | 0.000306 | 0.000794 |
| INSULIN_DON | deceased donor-was donor given insulin within 24 hrs pre cross clamp? | 1.153734 | 1.25167 | 1.221194 | 0.003801 | 0.008346 |
| LI_BIOPSY | cadaver donor liver biopsy | 0.408304 | 0.427617 | 0.383156 | 0.06162 | 0.092889 |
| LISTYR | actual year registrant listed (without date offset) | 2016.481 | 2013.626 | 2012.877 | ######## | ######## |
| MALIG_TCR | candidate any known malignancies @ listing | 0.231691 | 0.233853 | 0.239704 | 0.651569 | 0.707618 |
| MED_COND_TRR | recipient medical condition pre-transplant @ transplant | 2.498608 | 2.389755 | 2.444239 | 0.000298 | 0.000791 |
| MELD_PELD_LAB_SCORE | meld/peld lab score at time of transplant | 25.24101 | 26.16704 | 24.98797 | 0.039208 | 0.064779 |
| NON_HRT_DON | deceased donor-non-heart beating donor | 0.012347 | 0.020045 | 0.026377 | 1.12E-05 | 3.52E-05 |
| PERM_STATE | state of residency @ registration | 25.89808 | 26.2294 | 26.35308 | 0.41344 | 0.497112 |
| PH_DON | ddr:blood ph: | 7.402009 | 7.402561 | 7.398903 | 0.425939 | 0.506116 |
| PORTAL_VEIN_TCR | recipient history of portal vein thrombosis @ registration | 0.207602 | 0.233853 | 0.19528 | 0.598718 | 0.657288 |
| PREV_AB_SURG_TCR | recipient previous upper abdominal surgery @ registration | 1.055078 | 1.138085 | 1.118001 | 0.011471 | 0.022281 |

| | | | | | | |
|---|---|---|---|---|---|---|
| PRI_PAYMENT_TCR | recipient primary projected payment type @ registration | 2.04842 | 2.077951 | 2.099954 | 0.044646 | 0.071575 |
| PRI_PAYMENT_TRR | recipient primary payment source @ transplant | 2.136182 | 2.195991 | 2.207311 | 0.012626 | 0.024061 |
| PROTEIN_URINE | deceased donor protein in urine | 1.010774 | 0.926503 | 0.919019 | 0.000267 | 0.000729 |
| PT_DIURETICS_DON | deceased donor-diuretics b/n brain death w/in 24 hrs of procurement | 1.298753 | 1.314031 | 1.277186 | 0.591643 | 0.656658 |
| PT_STEROIDS_DON | deceased donor-steroids b/n brain death w/in 24 hrs of procurement | 1.442198 | 1.494432 | 1.505784 | 0.008509 | 0.017188 |
| PT_T4_DON | deceased donor-thyroxine-t4 b/n brain death w/in 24 hrs of procurement | 1.212444 | 1.327394 | 1.221657 | 0.051387 | 0.081096 |
| PULM_INF_DON | deceased donor-infection pulmonary source | 0.624016 | 0.547884 | 0.52522 | 4.48E-17 | 4.52E-16 |
| PX_STAT | recipient status(died, retx, lost, alive) | 0.101804 | 1 | 0.972235 | 0 | 0 |
| SGOT_DON | deceased donor-terminal sgot/ast | 83.13606 | 82.72428 | 82.35493 | 0.316884 | 0.410324 |
| SGPT_DON | deceased donor-terminal sgpt/alt | 85.67643 | 76.35635 | 80.92503 | 0.875757 | 0.902566 |
| TATTOOS | deceased donor-tatoos | 0.895654 | 0.788419 | 0.744563 | 6.06E-10 | 3.60E-09 |
| TBILI_DON | deceased donor-terminal total bilirubin | 0.8767 | 0.950379 | 0.913452 | 0.034557 | 0.061232 |
| TBILI_TX | recipient total bilirubin @ transplant | 7.645676 | 8.565768 | 7.650375 | 0.032561 | 0.058726 |
| TIPSS_TCR | recipient transjugular intrahepatic portacaval stint shunt(tipss) @ registration | 0.195376 | 0.273942 | 0.226284 | 0.005597 | 0.011536 |
| TRANSFUS_TERM_DON | ddr:number of transfusions during this (terminal) hospitalization: | 0.671953 | 0.552339 | 0.596946 | 0.035504 | 0.061826 |
| URINE_INF_DON | deceased donor-infection urine source | 0.145382 | 0.115813 | 0.137436 | 0.162897 | 0.225378 |
| VASODIL_DON | deceased donor-vasodilators w/in 24hrs pre-cross clamp | 0.301658 | 0.356347 | 0.297547 | 0.271688 | 0.361059 |
| WGT_KG_TCR | recipient weight (kg) @ registration | 95.71879 | 95.07509 | 94.21898 | 0.080345 | 0.119336 |
| WORK_INCOME_TCR | work for income at registration? | 0.447161 | 0.345212 | 0.315132 | 2.59E-10 | 1.64E-09 |
